# Ferroptosis and bone health: bridging the gap between mechanisms and therapy

**DOI:** 10.3389/fimmu.2025.1634516

**Published:** 2025-07-16

**Authors:** Renyan Xiao, Zhongyu Han, Peng Jia, Pei Li, Meng Gong, Yijin Cai, Lina Pang, Xiangyin Ye, Song Jin

**Affiliations:** ^1^ School of Health Preservation and Rehabilitation, Chengdu University of Traditional Chinese Medicine, Chengdu, China; ^2^ Zhongda Hospital, School of Medicine, Southeast University, Nanjing, China; ^3^ School of Acupuncture and Tuina, Chengdu University of Traditional Chinese Medicine, Chengdu, China; ^4^ School of Medical and Life Sciences, Chengdu University of Traditional Chinese Medicine, Chengdu, China; ^5^ Department of Rehabilitation, Hospital of Chengdu University of Traditional Chinese Medicine, Chengdu, China

**Keywords:** ferroptosis, bone microenvironment, bone and joint diseases, mechanisms, therapeutic prospects

## Abstract

Ferroptosis is a novel type of programmed cell death that was discovered in recent years and is closely associated with disorders in iron cycling, abnormal lipid metabolism, excessive intracellular reactive oxygen species, and cellular antioxidant-related signaling pathways. Numerous studies have shown that ferroptosis plays a critical role in the development and progression of bone and joint diseases, although the underlying mechanisms remain incompletely understood. This review aims to outline the relevant mechanisms of ferroptosis, its implications in the bone microenvironment, and the mechanisms of action and therapeutic perspectives of ferroptosis in common bone and joint diseases, with the goal of informing future clinical research and treatment strategies targeting ferroptosis under these conditions.

## 1 Introduction

Degenerative orthopaedic diseases, such as osteoporosis (OP), osteoarthritis (OA), and lumbar disc herniation (LDH), gravely undermine patients’ quality of life. They inflict pain, lead to functional impairment and disability, and generate a massive financial burden for families and society (1). Osteosarcoma (OS) is the most common type of primary malignant bone tumor; it frequently occurs at the metaphysis of long bones in adolescents and strongly affects their growth and development (2).

Ferroptosis was proposed as a novel form of cell death in 2012. It is distinct from traditional forms of programmed cell death, such as apoptosis and pyroptosis, in both biochemical and cellular morphological aspects (3). Ferroptosis is a type of programmed cell death that is driven by iron and is biochemically characterized by the significant accumulation of lipid reactive oxygen species (ROS) within cells. As redox-active trace elements, iron ions are essential regulators of multiple cellular functions. Under pathological conditions, the accumulation of excess iron and ROS can result in ferroptosis. Morphologically, it is primarily observed as the atrophy of mitochondria. The number of cristae in mitochondria may decrease or disappear. While investigations into ferroptosis across different domains are still in the preliminary phase, a growing body of research has recognized its crucial involvement in the pathological mechanisms of orthopaedic ailments. Therefore, comprehensively reviewing and analyzing the connections between ferroptosis and bone and joint diseases is essential.

This review seeks to clarify the molecular mechanisms underlying ferroptosis, its significance within the bone microenvironment, and the modes of action and potential therapeutic targets associated with ferroptosis in conditions such as OP, OA, OS, and LDH. Furthermore, the future prospects of targeting ferroptosis for the treatment of these disorders, as well as the current limitations and challenges within the field, are discussed.

## 2 Molecular mechanisms of ferroptosis

Ferroptosis, a novel form of cell death distinct from apoptosis, pyroptosis and autophagy, is an iron-dependent programmed form of cell death characterized by an iron metabolism disorder, imbalance of the redox system and the accumulation of lipid peroxides (4). During this form of cell death, a discrepancy exists between intracellular oxidative stress and the antioxidant defense system. This imbalance may lead to cell death by triggering lipid oxidation in the membrane, compromising membrane integrity, inducing lipid cross-linking or causing further oxidative damage to macromolecules. Ferroptosis was discovered a decade ago. However, similar cell death phenomena have been reported in previous scientific research. For example, ‘oxygen toxicity’ describes a form of cell death caused by excess oxidative stress in neuronal cells (5). In the mid-20th century, Harry Eagle et al. made a pioneering discovery that depriving cells of cystine could lead to cell death. They also found that cells with the ability to synthesize cysteine internally could resist ferroptosis (6, 7).

Ferroptosis is an evolutionarily conserved process that occurs in mammals and various organisms (such as plants and microorganisms). It has crucial impacts on the growth and diseases of the abovementioned organisms (8, 9). Ferroptosis involves a complex regulatory network and is regulated by various biological pathways. These pathways include cellular metabolism, such as the intracellular iron and lipid cycles, along with genes that regulate ferroptosis-associated protein expression (10).

### 2.1 Iron accumulation

**Figure 1:**
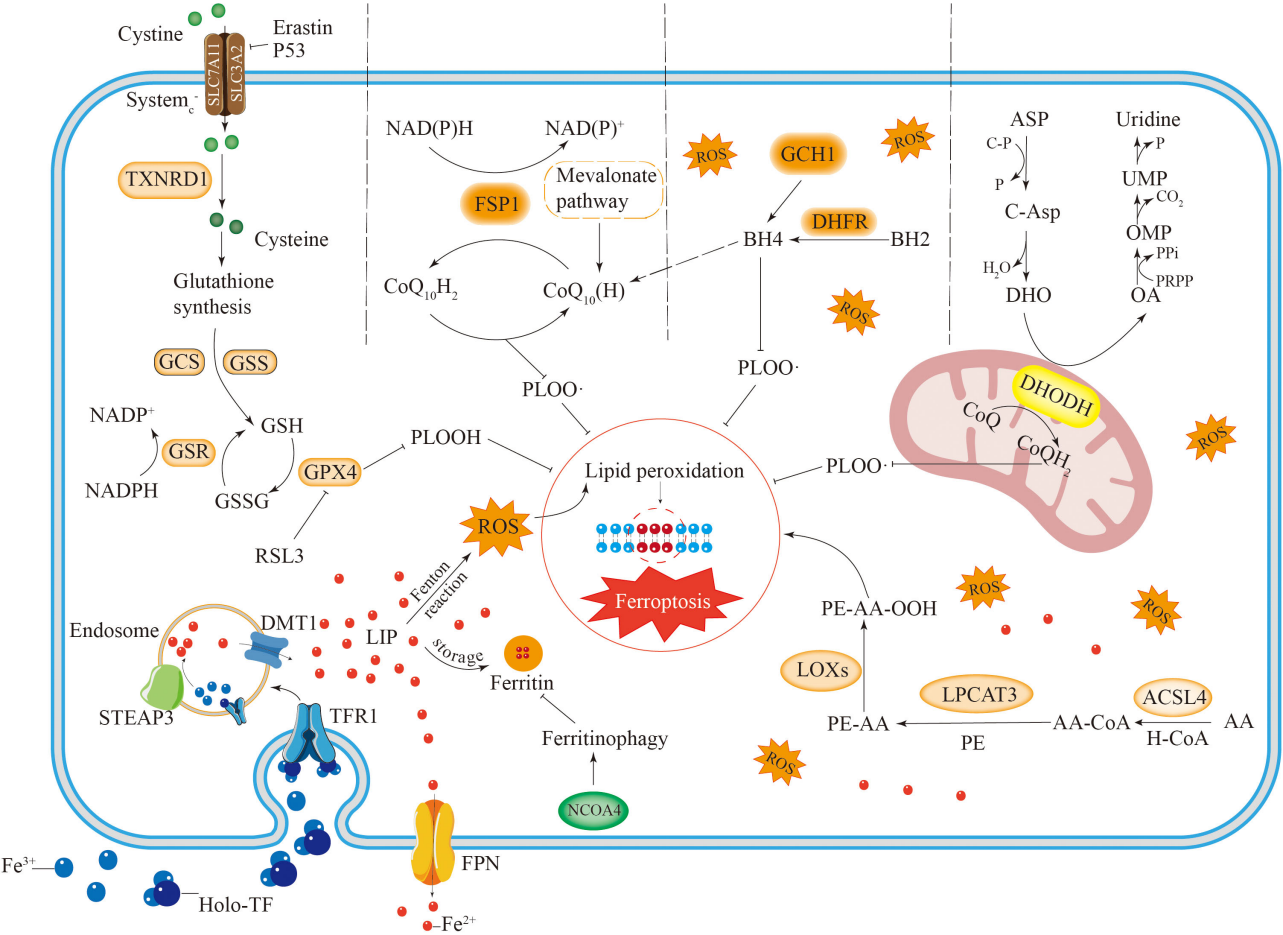
The occurrence and regulatory mechanisms of ferroptosis. Iron metabolism involves iron uptake, storage and export. Iron is mainly stored in cells in the form of free iron (LIP) and ferritin. Reactive iron can trigger the Fenton reaction, leading to the accumulation of ROS and inducing ferroptosis. Autophagy of ferritin mediated by NCOA4 can increase free iron in cells. AA undergoes a series of reactions to cause lipid peroxidation, leading to ferroptosis in cells. There are four antioxidant pathways in ferroptosis, including the System X_c_^–^GSH-GPX4 pathway, NADPH-FSP1-CoQ10 pathway, GCH1-BH4 pathway and DHODH-CoQH2 pathway. They inhibit lipid peroxidation to suppress ferroptosis in cells.

Iron is crucial for various life activities of cells (11). An imbalance in the iron redox reaction and the iron ion cycle in cells are important factors leading to ferroptosis in cells (12). Extracellular iron enters the cell by binding to transferrin (TF), which recognizes transferrin receptor 1 (TFR1) on the cell membrane (13). Within the cell, iron is reduced to its divalent form by the six-transmembrane epithelial antigen of the prostate 3 (STEAP3) and is then translocated into the cytoplasm by divalent metal transporter 1 (DMT1) (**Figure 1**) (14, 15). Intracellular iron is stored either as ferritin or in the labile iron pool (LIP). Iron subsequently exits the cell into the extracellular environment through the iron transport protein ferroportin (FPN) (16, 17).

Excessive free iron leads to iron accumulation, which triggers nonenzymatic, iron-dependent Fenton chain reactions that directly produce excessive reactive oxygen species, thereby causing oxidative damage to cells (18). Therefore, excessive iron accumulation promotes ferroptosis in cells. IRP1 and IRP2 are crucial proteins involved in the meticulous regulation of intracellular iron levels. These proteins play significant roles in managing the iron cycle within the cell by engaging in posttranscriptional regulation. This process specifically targets genes that are associated with iron transport inside the cell, ensuring that iron homeostasis is effectively and efficiently maintained (19, 20). Through their regulatory activities, IRP1 and IRP2 help control the availability of iron, which is essential for various cellular functions and overall cellular health. Ferritin, which is composed of a light chain (FTL) and a heavy chain (FTH), has significant antiferroptotic properties. The degradation of ferritin via lysosome-associated ferritin-induced autophagy leads to elevated levels of free reactive iron within cells and an accumulation of iron, which ultimately results in iron-induced cellular death (21, 22). Suppressing ferritin-induced autophagy triggered by nuclear receptor coactivator 4 (NCOA4) promotes the effective use of cellular iron ions, thus reducing the occurrence of cellular ferroptosis (**Figure 1**).

Poly (RC)-binding proteins (PCBPs), which are iron chaperones that transfer iron to corresponding proteins- can transport Fe^2+^ to ferritin, thereby increasing ferroptosis resistance in hepatocytes (23). Haem oxygenase 1 (HO-1) facilitates the breakdown of heme, resulting in increased levels of reactive iron ions within the cell, which play a role in inducing cellular iron-related death. Notably, HO-1 has both positive and negative regulatory effects on ferroptosis (24, 25). ATM, a serine–threonine protein kinase, can block metal - regulated transcription factor 1 (MTF1), thereby promoting ferroptosis (26). Furthermore, prominin 2 (PROM2) reduces ferroptosis in breast cancer cells by promoting the export of iron from these cells (27).

Moderate levels of cellular iron are essential for maintaining normal bone health. Bone remodeling and resorption during physiological processes require adequate amounts of iron. Abnormal iron metabolism plays a significant role in various diseases. In the pathology of OA, cellular iron overload can increase localized inflammatory mediators, contributing to OA development (28). Additionally, abnormal iron metabolism can impair osteoclast function and induce oxidative stress, leading to an imbalance in bone homeostasis and bone loss, which is associated with OP formation (29, 30). Iron overload in chondrocytes can elevate markers of chondrocyte catabolism, triggering cartilage degeneration. Furthermore, iron overload in synoviocytes can influence cytokine expression, resulting in synovial inflammation linked to OA formation (28, 31). Therefore, iron metabolism in the bone microenvironment significantly impacts both physiological processes and the pathological development of bone.

### 2.2 Lipid peroxidation

A disruption of cell membrane integrity, which can lead to cell death, represents one potential mechanism through which ferroptosis occurs. Polyunsaturated fatty acids (PUFAs) play a critical role as key constituents of the cytoplasmic membrane and act as major targets of ROS (32–34). As a result, managing lipid metabolism is essential to the regulatory processes related to ferroptotic cell death. The enzyme adenylate-activated protein kinase (AMPK), which acts as a sensor of cellular energy levels, influences ferroptosis by facilitating the phosphorylation of acetyl coenzyme A carboxylase (ACC) and the production of polyunsaturated fatty acids (35). Additionally, the dissection of its upstream kinase, liver kinase B1 (LKB1), increases the sensitivity of mouse embryonic fibroblasts to ferroptosis (36).

In the process of ferroptosis, PUFAs serve as major targets for lipid peroxidation. Research has shown that oleic acid can effectively prevent erastin-triggered ferroptosis by engaging in competition with PUFAs to be incorporated into PLs (37). PUFAs can be integrated into cellular membranes via acylcarnitine synthase 4 (ACSL4) (38) and lysophosphatidylcholine acyltransferase 3 (LPCAT3) (39), increasing cellular vulnerability to ferroptosis. The oxidation of unsaturated fatty acids may proceed through both enzymatic and nonenzymatic mechanisms. For example, arachidonic acid (AA) is converted by ACSL4 and LPCAT3. Subsequently, the resultant molecule undergoes oxidation through either lipoxygenase (Lox) or a non-enzymatic pathway, leading to the formation of PE-AA-OOH (**Figure 1**) (40, 41).

Significant lipid peroxidation plays a crucial role in ferroptosis, potentially resulting in cellular death via various mechanisms. These mechanisms involve modifications to the membrane’s lipid bilayer structure, the creation of membrane pores that impair barrier function, a decrease in membrane thickness, and alterations in permeability (42). Furthermore, peroxidated lipids can be decomposed into toxic derivatives, which may induce significant cytotoxicity (43, 44).

Lipid peroxidation serves as a crucial mechanism in ferroptosis, and its occurrence within the bone microenvironment significantly contributes to cellular ferroptosis, thereby impacting bone health. Inhibiting lipid peroxidation in bone microenvironment cells may represent a promising therapeutic approach for treating bone-related diseases. Notably, the inhibition of ACSL4 has been shown to reduce the incidence of neuronal ferroptosis, indicating that ACSL4 could be a viable target for the treatment of bone-related disorders (45). Furthermore, compounds that inhibit the lipid peroxidation process, such as baicalein and zileuton, have demonstrated the ability to enhance bone density by promoting bone formation, suggesting that these agents may serve as potential therapeutic options for OP (46).

### 2.3 Antioxidant mechanisms in ferroptosis

System X_C_^–^GSH–GPX4 pathway is an important antioxidant pathway in cells. Glutathione peroxidase 4 (GPX4) is a key enzyme characterized by the presence of selenocysteine, which plays a vital role in neutralizing phospholipid hydroperoxides (47). Research has shown that when GPX4 is absent, lipid peroxidation leads to non-apoptotic cell death in mouse embryonic fibroblasts (48). Both the expression levels and functionality of GPX4 are influenced by selenium and glutathione (GSH). Selenium participates in the process of GPX4 synthesis and substitutes for the sulfur in cysteine (49). Furthermore, selenium can also induce the upregulation of GPX4 expression through the transcriptional pathway to reduce ferroptosis (50).

GPX4 exerts its physiological functions through GSH. During the process of phospholipid hydroperoxide being reduced to the corresponding phospholipid alcohols, GSH acts as an electron donor, while glutathione disulfide (GSSG) is produced simultaneously (51). Glutathione disulfide reductase (GSR) can recycle oxidized glutathione using electrons sourced from reduced NADPH to restore glutathione (**Figure 1**) (52).

In the cytoplasm, GSH is synthesized through the catalytic action of glutamate cysteine ligase (GCL) and glutathione synthetase (GSS) (52). In this synthesis reaction, cysteine serves as the critical rate-limiting factor (53). Cysteine enters cells in its oxidized form, known as cystine, through System X_C_^-^. Once inside the cell, cystine is reduced to cysteine by the enzyme thioredoxin reductase 1 (TXNRD1) (**Figure 1**) (54). Furthermore, cysteine may also be synthesized from methionine via the transsulfuration pathway (55). The reverse transporter protein System X_C_^-^, which operates as a heterodimeric complex of SLC7A11 and SLC3A2, enables the exchange of cystine and glutamate across the plasma membrane (3, 56). P53 can induce ferroptosis and consequently tumor cell death by inhibiting GSH synthesis through the downregulation of SLC7A11 expression (57). Erastin can directly inhibit cystine uptake, leading to ferroptosis, whereas RSL3 can inhibit GPX4 to induce ferroptosis (**Figure 1**) (58).

Ferroptosis inhibitory protein 1 (FSP1) serves as an important antioxidant involved in the mechanism of ferroptosis (59). By utilizing NADPH, FSP1 reduces coenzyme Q to ubiquinol, effectively halting lipid autoxidation (**Figure 1**) (60, 61). Moreover, FSP1 can inhibit cell death through α-tocopherol-mediated antioxidation, which is more potent than the former mechanism (62). The antioxidant effects of the FSP1 pathway are primarily mediated by coenzyme Q10 (CoQ10), an isoprenoid benzoquinone compound (63, 64). Additionally, panthenol (CoQ10H2), serves as an important antioxidant that targets free radicals. It exerts its antiferroptotic effect by directly scavenging lipid peroxidation-inducing free radicals (65).

CoQ10 can be synthesized by the mevalonate (MVA) pathway using acetyl coenzyme A (66). Supplementation with farnesyl pyrophosphate, a product of CoQ10 synthesis, has been shown to inhibit ferroptosis induced by FIN56 (67), highlighting the endogenous inhibitory role of CoQ10 in this process. In addition to its redox enzyme function, FSP1 also has a membrane repair function. It can suppress ferroptosis by triggering the ESCRT-III pathway for membrane repair (55, 68). These findings suggest that FSP1 cannot protect against ferroptosis triggered by the deletion of GPX4. This finding indicates that the NADPH–FSP1–CoQ10 pathway is a separate and parallel system. It functions in conjunction with GPX4 and GSH to inhibit lipid ROS and ferroptosis (60, 61).

Tetrahydrobiopterin (BH4) possesses robust antioxidant properties, enabling it to directly curb lipid peroxidation (69, 70). Additionally, BH4 participates in coenzyme Q10 synthesis, shielding cells from ferroptosis (**Figure 1**). In addition, BH4 functions as a cofactor for several key enzymes related to dopamine and NO production. Research has demonstrated that both dopamine and NO are associated with ferroptosis (71).

In the synthesis of BH4, GTP cyclohydrolase 1 (GCH1) serves as the key regulatory enzyme, thereby determining the level of BH4 (**Figure 1**). Moreover, BH4 can be regenerated by dihydrofolate reductase (DHFR). BH4 inhibits ferroptosis by selectively preventing the depletion of phospholipids from two polyunsaturated fatty acid tails (69). A previous study confirmed that dopamine can inhibit ferroptosis induced by erastin (72) and that BH4 is an auxiliary factor for the key enzyme that regulates dopamine synthesis. Additionally, a positive correlation was observed between the expression of GCH1 and the levels of BH4 in cells. Elevated intracellular levels of BH4 inhibit lipid peroxidation and prevent cellular ferroptosis. GCH1, independent of the GPX4 antioxidant pathway, primarily enhances the antioxidant activity of cells by promoting BH4 production and thus protects cells from ferroptosis. The GCH1–BH4–DHFR pathway clearly serves as a key regulatory pathway for ferroptosis. Furthermore, other antioxidant agents, such as vitamin E (73), thioredoxin (74), and aldehyde–ketone reductase family 1, also play certain roles in ferroptosis (75).

Dihydroorotic acid dehydrogenase (DHODH) is a mitochondrial enzyme that contains iron and depends on flavin; it is located in the inner membrane of mitochondria. The primary role of this enzyme is to facilitate the fourth step in pyrimidine synthesis by converting dihydroorotate (DHO) into orotate (**Figure 1**). At the same time, it reduces ubiquinone to dihydroubiquinone via electron transfer across the mitochondrial inner membrane (76). CoQH2 acts as an antioxidant that traps free radicals, thereby inhibiting lipid peroxidation at the inner mitochondrial membrane and ultimately preventing iron-induced cell death (**Figure 1**) (77).

An experimental analysis of a group of cancer cell lines with low and high GPX4 expression demonstrated that inhibiting DHODH could induce ferroptosis in tumor cells with low GPX4 expression while enhancing ferroptosis resistance in cancer cells with high GPX4 expression. The DHODH–CoQH2 pathway functions as an antioxidant pathway that works independently of the GPX4 system, providing an antiferroptotic effect by preventing mitochondrial lipid peroxidation (77, 78).

The antioxidant system of cells serves as a critical defense mechanism against ferroptosis. Research has demonstrated that decreased levels of GPX4 and SLC7A11 in the bone tissue of mice with OP correlate with increased bone mineral density and improved bone quality upon elevation of glutathione levels (79). Furthermore, reduced GPX4 levels in chondrocytes heighten their susceptibility to ferroptosis, adversely impacting normal cartilage function (80). These findings suggest that ferroptosis, induced by an imbalance in the antioxidant system, may significantly influence bone health. Consequently, targeting ferroptosis through the activation of the cellular antioxidant system represents a promising avenue for intervention in bone diseases. Furthermore, inducing ferroptosis by inhibiting the cellular antioxidant system presents a potential therapeutic strategy for OS, as various anticancer agents exert their effects by targeting antioxidant system-related proteins such as GPX4 (57).

## 3 Ferroptosis in bone microenvironment

The bone microenvironment encompasses the local setting where bone tissue resides, comprising key cellular components such as osteoblasts, osteoclasts, and osteocytes within the tissue, along with adipocytes, vascular networks, immune cells, and an abundance of bone marrow and extracellular matrix (81). The bone microenvironment is important for maintaining normal bone physiology and regulating it in pathological settings. Ferroptosis is intricately linked to the bone microenvironment, and the ferroptosis of cells within this microenvironment significantly contributes to various orthopaedic diseases. Ferroptosis of osteoblasts and osteoclasts affects bone growth and repair. Furthermore, a normal bone immune microenvironment is vital for maintaining bone health, and a potential connection exists between immune cell metabolism and the ferroptosis network. Ferroptosis can modulate immune cell function. In particular, the occurrence of this process in immune cells impacts both their numbers and effectiveness, whereas in nonimmune cells, ferroptosis can initiate immune responses via damage-associated molecular patterns.

### 3.1 Ferroptosis in osteoclasts

Normal iron cycling in cells plays an important regulatory role in maintaining bone health (82). Osteoblasts form bone tissue, while osteoclasts absorb it. The two work in concert in bone formation and remodeling. Increasing evidence suggests that disturbances in iron metabolism can negatively influence the formation and functions of osteoblasts and osteoclasts, thereby disrupting the balance between osteogenesis and bone resorption. Consequently, maintaining iron homeostasis is vital for optimal bone growth and development (83).

During osteoclast differentiation, the demand for iron increases, and the expression of TFR1 is increased through posttranscriptional regulation, thereby increasing iron uptake by the cell. These findings demonstrate the positive regulatory effect of TFR1 on osteoclasts (84). FPN regulates cellular iron efflux (16, 17) and inhibits osteoclast maturation through pathways such as the JNK pathway. During the initial stages of osteoclast maturation, a reduction in the transcription of FPN promotes osteoclast differentiation (85). A study showed that increased plasma iron levels in FPN mutant mice led to decreased osteogenesis, indicating a negative regulatory role of ferroportin in osteoclasts (86, 87).

Osteoclasts, which are specialized cells responsible for bone resorption, arise from the differentiation of myeloid progenitor cells found in the bone marrow (88). The signaling pathway involving receptor activator of nuclear factor-κB ligand (RANKL), its receptor (RANK), and osteoprotegerin (OPG) plays a pivotal role in the regulation of bone resorption. This pathway is essential because it modulates the differentiation and activation of osteoclasts, thereby influencing the overall process of bone resorption (89, 90). Through this intricate signaling mechanism, the balance of bone remodeling is maintained, highlighting the importance of regulating iron levels to ensure optimal bone health.

Osteoclasts require substantial amounts of mitochondrial energy to perform their physiological functions, and iron ions are crucial for mitochondrial synthesis. Iron ion uptake mediated by TFR1 plays a significant regulatory role in osteoclast differentiation (91, 92). It regulates osteoclasts and mitochondria through the activation of the Src–Rac1–WAVE regulatory complex pathway, which in turn modulates mitochondrial respiration (93).

**Figure 2:**
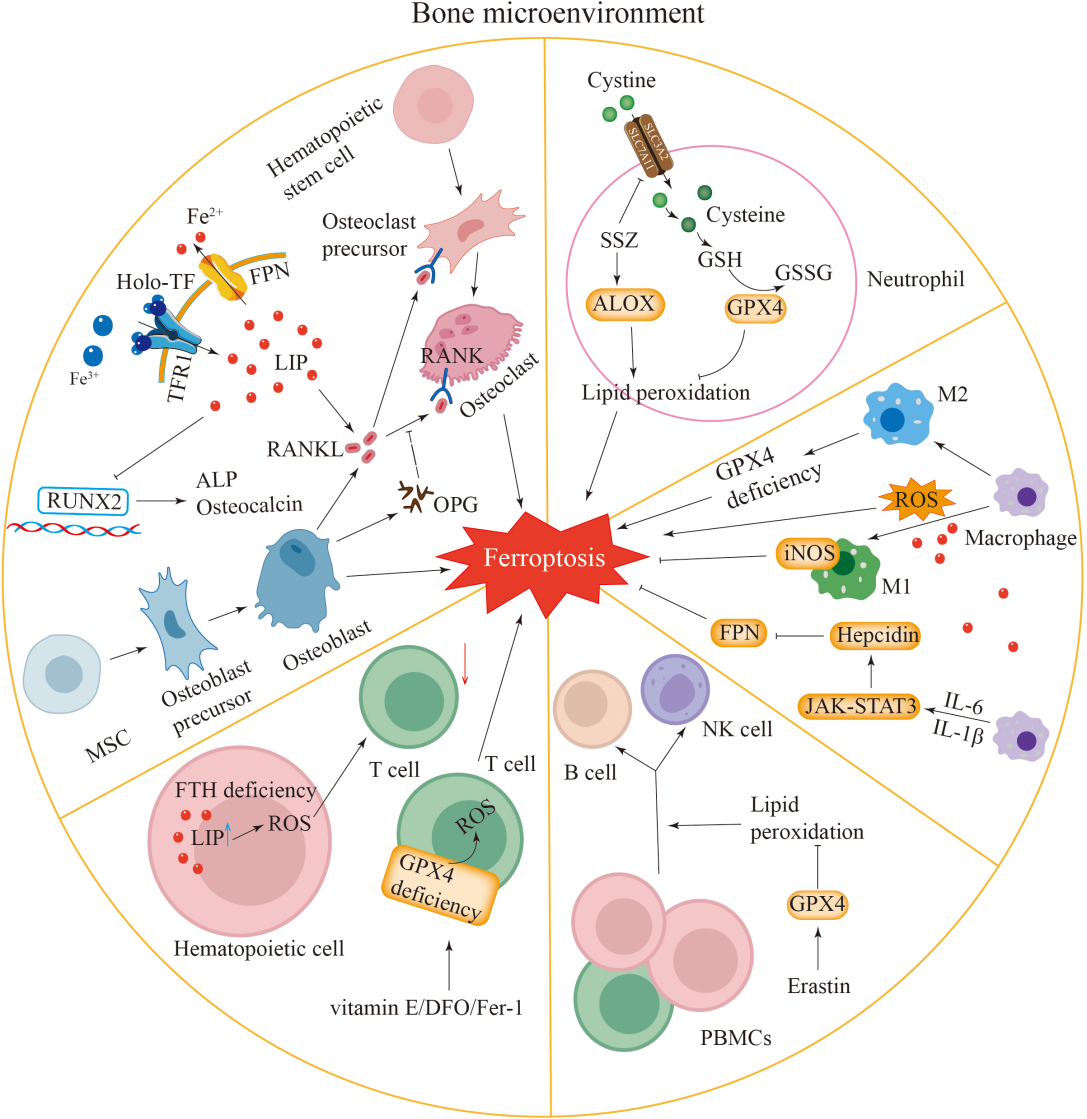
Ferroptosis in the bone microenvironment. Osteoblasts and osteoclasts: iron overload in osteoblasts and osteoclasts induces ferroptosis in these cells. Iron overload inhibits the expression of Runx2, ALP and osteocalcin, thereby affecting osteoblast function. Osteoblasts release RANKL to promote the differentiation of osteoclasts. Enhanced iron uptake mediated by TFR1 leads to an increased RANKL/OPG ratio, promoting osteoclastogenesis. Neutrophils: the expression of GPX4 in neutrophils is inhibited, which can induce ferroptosis. SSZ can promote ferroptosis by inhibiting SLC7A11 and activating ALOX. Macrophages: macrophages have iron overload. Macrophages can promote ferroptosis by generating ROS, and ROS can also promote the transformation of macrophages into M1-type macrophages. Macrophages can activate the JAK-STAT3 pathway by secreting IL-6 and promote the transcription of hepcidin by secreting IL-1β, leading to a decrease in FPN expression and causing ferroptosis. The expression of iNOS in M1-type macrophages inhibits ferroptosis. The loss of GPX4 activity in M2-type macrophages leads to ferroptosis. Lymphocytes: the deletion of H-ferritin gene in hematopoietic cells increases reactive oxygen species and active iron, resulting in a reduction in T cell numbers. Vitamin E, DFO, and Fer-1 can rescue the ferroptosis of GPX4-deficient T cells. Erastin can promote the differentiation of PBMCs into B cells and NKs.

When iron levels are excessively high, the RANKL/OPG ratio increases, leading to the promotion of osteoclast maturation and bone resorption through TFR1-mediated iron uptake (**Figure 2**). Conversely, iron-chelated lactoferrin inhibits osteoclast-mediated bone resorption by reducing the RANKL/OPG ratio, thereby increasing the bone mineral density (92, 94). TFR1-mediated iron uptake mainly regulates the growth and development of mature osteoclasts, but its impact on osteoclast precursors is relatively limited (93). These findings suggest that iron overload might lead to bone loss by promoting the activity and function of osteoclasts (95, 96).

### 3.2 Ferroptosis in osteoblasts

Osteoblasts, which are essential for bone development, arise from mesenchymal progenitor cells that are vital for managing the processes of bone creation and remodeling (97). Iron primarily exerts its influence by inhibiting the expression of alkaline phosphatase (ALP) and the mineralization of osteoblasts. Notably, trivalent iron has a more pronounced inhibitory effect than does divalent iron (98). Alkaline phosphatase is one of the key factors regulating the mineralization of osteoblasts. The decrease in ALP activity could strengthen the inhibitory effects of Fe^3+^ and Fe^2+^ on osteoblasts.

Iron hinders the function of osteoblasts by reducing the expression levels of markers associated with osteogenic differentiation in both C2C12 myoblasts and bone marrow-derived mesenchymal stem cells (BM-MSCs) (99, 100). These findings indicate that iron has a greater inhibitory effect on osteoblast precursors than on mature osteoblasts during osteogenic induction, which is similar to the regulation of osteoclasts by TFR1. Excessive iron accumulation may suppress the function and osteogenic differentiation of osteoblasts. Research has indicated that when iron ions are present at specific concentrations, they can inhibit the activity of Runt-related transcription factor 2 (Runx2), a critical regulator of osteoblast differentiation (**Figure 2**). This inhibition results in reduced expression of key osteogenic markers, such as alkaline phosphatase (ALP) and osteocalcin, which are essential for the proper differentiation of bone marrow-derived mesenchymal stem cells (BM-MSCs). Moreover, the excessive accumulation of intracellular iron ions further contributes to the downregulation of the osteoblast phenotype, which is essential for maintaining healthy bone formation (101). Thus, iron overload is inferred to cause bone damage by promoting osteoclast activity while simultaneously suppressing the formation of osteoblasts.

### 3.3 Ferroptosis in neutrophils

Neutrophils are intricate cells with many specialized functions that are vital for managing different pathophysiological processes. Serving as effector cells in the innate immune response, these cells represent the most abundant type of immune cells among human white blood cells. The primary bactericidal role of neutrophils is accomplished via phagocytosis and the creation of neutrophil extracellular traps (NETs), which are composed of degraded chromatin and granular proteins from within the cell, triggered by various stimuli. Even after the death of neutrophils, NETs can still perform their bactericidal function.

Ferroptosis may be involved in the formation of NETs and the regulation of neutrophil recruitment (102). The ferroptosis inhibitor sulfasalazine (SSZ) enhances ferroptosis by blocking SLC7A11 and stimulating the lipoxygenase ALOX (**Figure 2**) (103). Research indicates that ether-bonded glycerolipids increase cellular sensitivity to ferroptosis, whereas ether lipids are crucial for the formation of the extracellular meshwork induced by sulfasalazine (104). This result suggests a potential association between these two phenomena. Furthermore, ferroptosis is related not only to NETs but also to autoimmune diseases associated with neutrophils. Nevertheless, the impact of neutrophil ferroptosis on the progression of these diseases has yet to be completely verified.

### 3.4 Ferroptosis in macrophages

Macrophages stem from monocytes and can be divided into two types: M1 and M2. They contribute to both innate and adaptive immunity in vertebrates. Phagocytes primarily function in phagocytosis, which involves the engulfment and digestion of cell debris and pathogens, either as fixed or free-floating cells.

Macrophages are closely associated with ferroptosis. Ferroptotic and M1-type macrophages both exhibit iron accumulation. Excess iron may promote the polarization of macrophages towards the M1 phenotype (**Figure 2**). In terms of cytokines, macrophages cause inflammation by releasing proinflammatory factors. Similarly, an increase in the levels of proinflammatory factors also occurs within ferroptotic cells. By releasing cytokines, macrophages can regulate the activity of Lox, thereby inducing ferroptosis. Inducible nitric oxide synthase (iNOS) plays a crucial role in M1 macrophages by negatively regulating ferroptosis (**Figure 2**) (105). An impairment of the GPX4 system in M2-type macrophages can lead to cellular ferroptosis (106). Macrophages are capable of generating ROS, which in turn can drive their differentiation into M1-type macrophages (107, 108). IL-6 secreted by macrophages promotes hepcidin transcription via the activation of the JAK-STAT3 signaling pathway (**Figure 2**). Furthermore, macrophages secrete IL-1β to upregulate hepcidin expression. These combined actions result in decreased levels of FPN expression, causing an accumulation of intracellular iron and eventually resulting in iron-induced cell death (109, 110).

### 3.5 Ferroptosis in lymphocytes

Lymphocytes represent the smallest category of white blood cells. They are essential for the body’s immune response. These cells can be divided into three primary types according to their functions: T cells, B cells, and natural killer (NK) cells.

Maintaining proper iron balance within cells is essential for T-cell survival and the performance of their typical physiological roles (111). Elevated levels of ROS and iron in haematopoietic cells lacking the H-ferritin gene lead to a reduction in T-cell populations (**Figure 2**) (112). T cells deficient in GPX4 undergo ferroptosis, which results in compromised immune function (113). Research has shown that antioxidant interventions, including vitamin E, deferoxamine (DFO), or Fer-1, can alleviate ferroptosis in T cells lacking GPX4 (**Figure 2**) (114). Additionally, antibodies produced by B cells can influence iron-induced cell death, while ROS also impact the B-cell quantity and normal function (115, 116). Lipid peroxidation induced by the ferroptosis inducer erastin increases the proliferation of human peripheral blood mononuclear cells (PBMCs) and their differentiation into B cells and NKs (116).

### 3.6 Ferroptosis in other cells

BMSCs that differentiate into osteoblasts or chondrocytes are crucial for the development, reconstruction, and tissue regeneration of bone and cartilage. Iron homeostasis is of vital importance for their proliferation and differentiation.

Studies have confirmed that iron homeostasis imbalance severely damages the bone marrow microenvironment of mice, leading to bone destruction (117). Excessive iron can also trigger oxidative stress, leading to trabecular bone damage and bone loss. Antioxidants can inhibit the formation of osteoclasts and thereby alleviate this process (30). Furthermore, the accumulation of iron in synoviocytes disrupts the balance of various inflammatory factors in the joint, impairing the normal physiological function of cartilage. It increases the capacity of monocytes and synovial fibroblasts within the joint to absorb iron. This process accelerates the connective tissue degradation mediated by histone proteases and consequently induces joint disease (31, 118).

## 4 Ferroptosis in specific diseases

Ferroptosis is a type of cell death characterized by the accumulation of lipid reactive oxygen species inside cells. Presently, Ferroptosis is linked to a variety of diseases, including stroke, neurodegenerative disorders, cancers, and ischaemia–reperfusion injury (58). Recent studies have revealed that ferroptosis is also a key factor involved in the pathological mechanisms of bone and joint diseases. Ferroptosis exacerbates osteoporosis, triggers osteoarthritis, aggravates lumbar disc herniation, and is a promising target for osteosarcoma therapy.

### 4.1 Ferroptosis in osteoporosis

Osteoblasts form bone, and osteoclasts absorb bone. The two work in concert to maintain normal bone metabolism. Osteoporosis is a systemic disease characterized by a decreased bone density, destruction of the bone microstructure, and increased bone fragility (119). Osteoporosis can be divided into primary osteoporosis (POP) and secondary osteoporosis (120). Osteoporosis causes an increase in bone fragility, often leading to fractures in elderly and postmenopausal women (121, 122). Statistical data indicate that approximately thirty-three percent of older females and twenty percent of older males are affected (123).

Disrupted calcium metabolism may lead to osteoporosis, and an increasing body of research indicates a potential link between iron-induced cell death and osteoporosis (124). Intracellular iron overload not only inhibits osteoblast function but also enhances osteoclast activity, thereby contributing to osteoporosis. A significant amount of research has indicated that individuals with anaemia frequently experience osteoporosis as well (125, 126), which may be related to iron overload-mediated ferroptosis caused by long-term blood transfusions in anaemia patients (127), and their fracture risk is associated with the frequency of blood transfusions (128). Furthermore, research has revealed a link between haemochromatosis and osteoporosis. In individuals with haemochromatosis, osteoporosis is linked to iron overload (129), and ferroptosis might be the primary factor driving osteoporosis in these populations (130).

Elevated concentrations of reactive iron ions in osteoblasts and osteoclasts result in cellular iron toxicity. With the ongoing accumulation of intracellular iron ions, a notable increase in the levels of iron cycling phase proteins, including DMT1 and TFR1, occur within both osteoblasts and osteoclasts (131, 132). Consequently, iron overload-mediated ferroptosis contributes to the development of osteoporosis through its detrimental effects on osteoblasts, osteoclasts, and BM-MSCs, ultimately disrupting bone homeostasis.

**Figure 3:**
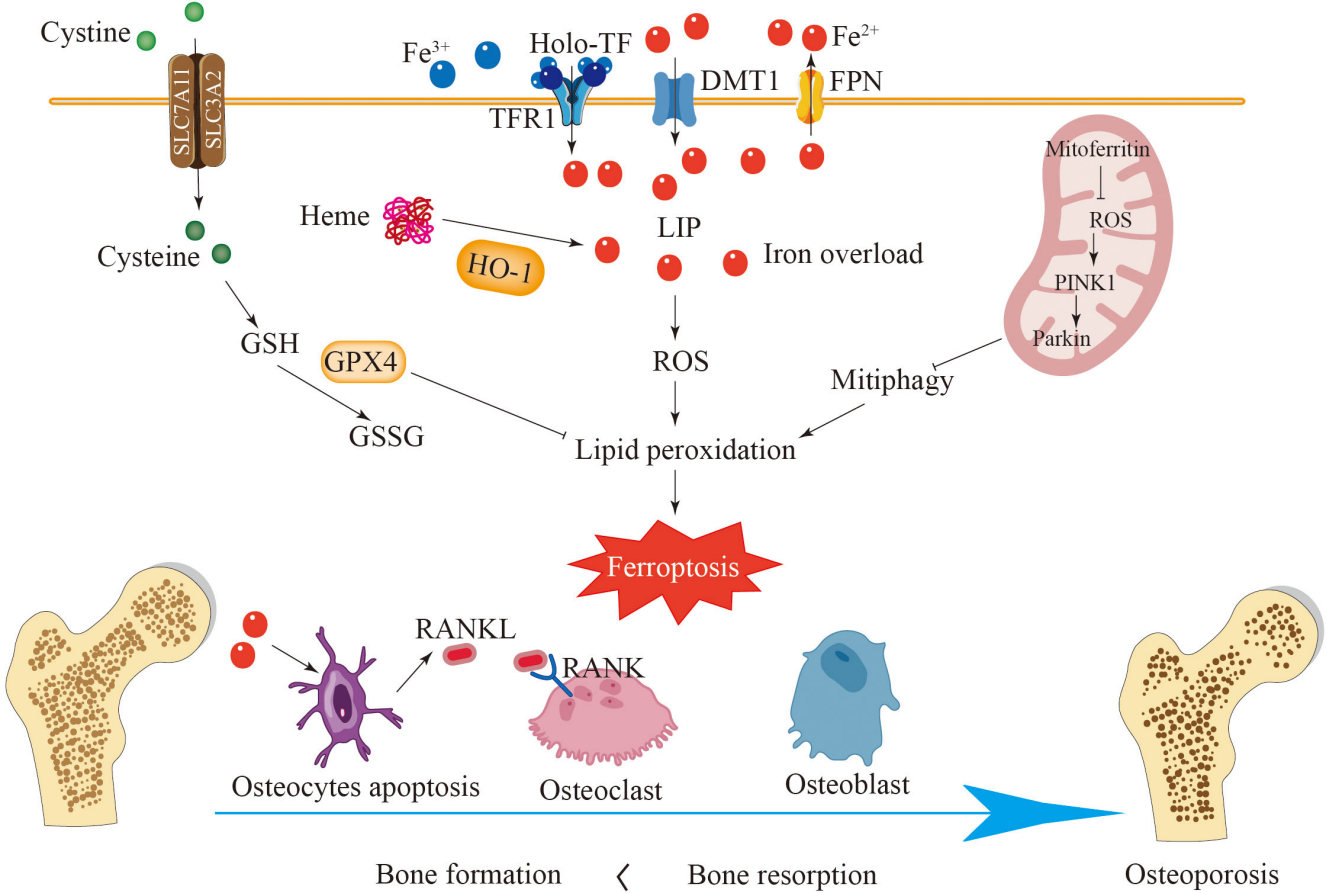
Ferroptosis plays a key role in the development of osteoporosis. In healthy bones, bone formation mediated by osteoblasts and bone resorption mediated by osteoclasts are in balance. Iron overload enhances the expression of DMT1 and TFR1 in osteoblasts and reduces the expression of SLC7A11 and GPX4, leading to ferroptosis of osteoblasts and disruption of bone formation. Iron overload can cause ferroptosis in osteoclasts and osteocytes and promote the secretion of RANKL, promoting the generation of osteoclasts. HO-1 catalyzes the degradation of heme to release a large amount of active iron, accelerating cell ferroptosis. Ferroptosis disrupts the balance between bone formation and bone resorption, leading to osteoporosis.

Osteoporosis is a prevalent complication of type II diabetes mellitus, with studies confirming a strong link between iron-mediated cell death and glucose metabolism. Increased serum ferritin levels have been observed in diabetic mice with osteoporosis, accompanied by significantly reduced levels of SLC7A11 and GPX4 in bone tissue (133). High glucose levels are capable of decreasing osteocalcin expression, reducing alkaline phosphatase activity, and suppressing bone mineralization (134, 135). Research has shown that the expression of HO-1 in osteocytes in a high-glucose microenvironment increases. HO-1 facilitates the breakdown of heme, resulting in the release of significant quantities of reactive iron ions, which then promote the Fenton reaction, ultimately producing lipid peroxides and causing ferroptosis in osteoblasts (**Figure 3**) (124, 136). This process suggests that HO-1 might serve as a potential therapeutic target for treating diabetic osteoporosis.

Ferroptosis is distinct from apoptosis; however, these two modes of cell death are not entirely independent in the pathological progression of OP. The cytokines involved in both processes interact with one another, contributing to the development of OP. Research has demonstrated that among the pathogenic factors associated with osteoporosis, advanced glycosylation end products can induce both ferroptosis and apoptosis in osteoblasts, ultimately leading to OP. Furthermore, DFO has been shown to inhibit this process (137). These findings suggest that targeting the shared key factors of apoptosis and ferroptosis may provide a more effective approach for the treatment of OP.

Mitoferritin located in mitochondria can reduce free iron in mitochondria and thereby decrease the sensitivity of cells to ferroptosis. The overexpression of mitochondrial ferritin has been shown to inhibit oxidative stress, which in turn mitigates ferroptosis (138). In contrast, decreased expression of mitochondrial ferritin can initiate mitochondrial autophagy through the ROS/PINK1/Parkin pathway, leading to the accumulation of ferritin and the enhancement of ferroptosis in osteoblasts (**Figure 3**) (135). These results suggest that mitochondrial ferritin could represent a promising future avenue for treating diabetic osteoporosis. Based on these findings, iron-induced cell death in osteoblasts is significantly involved in the progression of osteoporosis. Additionally, the pathway associated with iron-mediated death in osteoblasts, which utilizes various mechanisms, may represent a potential approach for the clinical management of osteoporosis.

Research on ferroptosis in OP is on the rise; however, the epigenetic regulatory mechanisms involved remain under investigation. It has been proposed that METTL14-mediated m6A modification plays a crucial role in the expression of GPX4. This indicates that the regulation of ferroptosis in osteoporosis via m6A modification may represent a promising avenue for future treatment strategies for OP (139).

### 4.2 Ferroptosis in osteoarthritis

Osteoarthritis is a degenerative condition of the joints characterized by the breakdown of cartilage, inflammation of the synovium, and changes in the subchondral bone structure (140, 141). Factors such as age, oestrogen levels, and mechanical stress play roles in increasing the likelihood of developing this disease (142–144). Osteoarthritis is characterized mainly by joint pain, deformity and functional impairment (145, 146). Additionally, it substantially increases the risks of cardiovascular incidents and overall mortality (147, 148). Currently, the number of OA patients worldwide exceeds 300 million (149).

Recent research has demonstrated a significant link between ferroptosis and OA, suggesting that this form of cell death might exacerbate the pathological mechanisms associated with OA (150, 151). Furthermore, the inhibition of ferroptosis represents a novel therapeutic target for OA management (152, 153). Iron overload has been observed in OA patients. Iron overload affects the progression of OA. From an imaging perspective, increased ferritin levels can exacerbate the severity of osteoarthritis (154, 155). Additionally, a positive correlation has been observed between serum iron levels and transferrin saturation and the progression of OA (156). A study conducted on guinea pigs susceptible to osteoporosis, which were placed on a diet low in iron, revealed that lower systemic iron levels delayed the development of cartilage damage. In contrast, when exogenous iron was administered, these animals developed knee osteoarthritis (157). These results highlight the significant impact of iron overload, stemming from iron dysregulation, on the development of OA.

**Figure 4:**
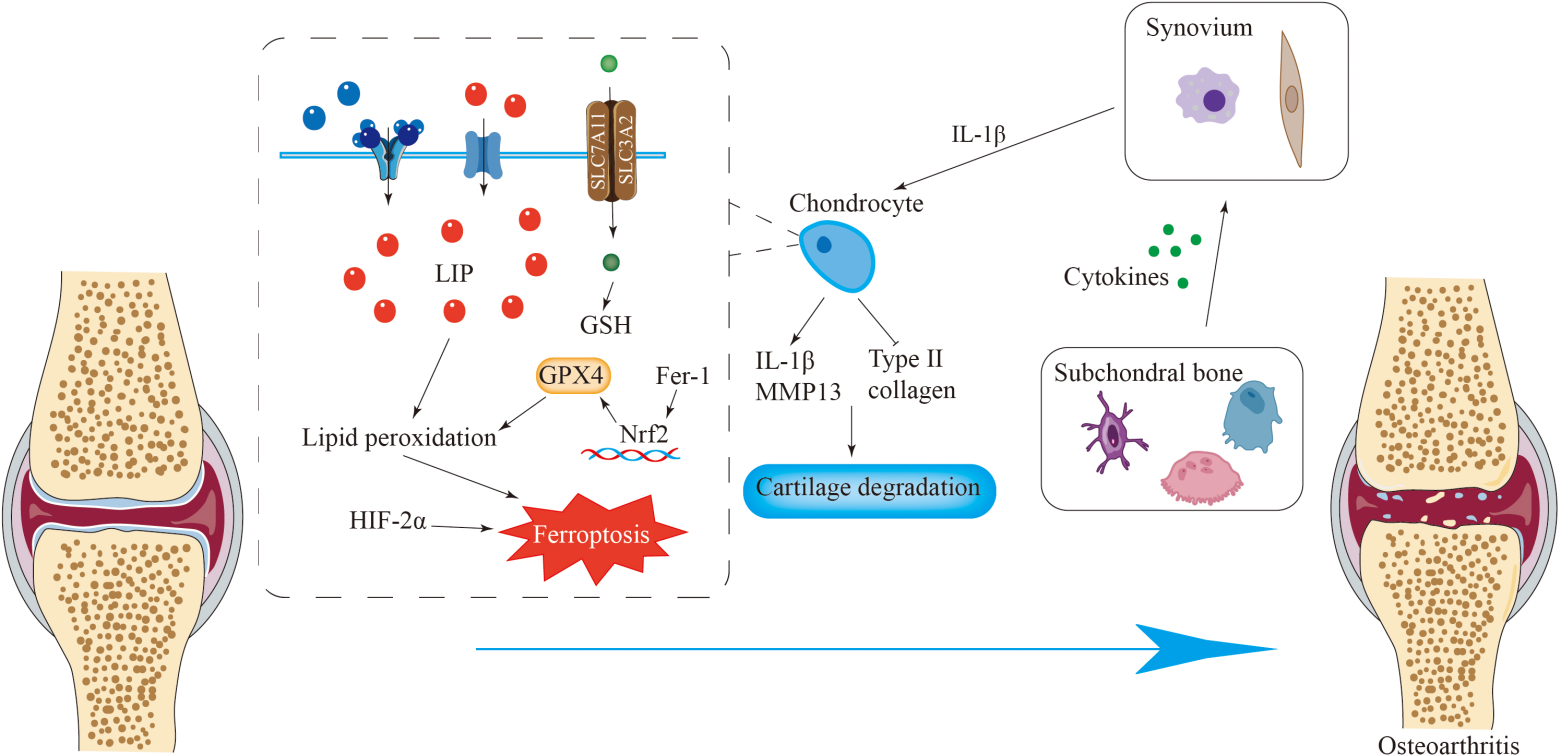
Ferroptosis is closely related to osteoarthritis. Iron overload in chondrocytes causes ferroptosis of chondrocytes, resulting in the destruction of the integrity of the extracellular matrix and the loss of chondrocytes. Fer-1 can activate the Nrf2 antioxidant system to counteract ferroptosis. Various cytokines released by cells such as osteoclasts in subchondral bone promote the release of pro-inflammatory cytokines by synovial cells, inducing ferroptosis of chondrocytes and ultimately leading to osteoarthritis.

Chondrocytes are involved in maintaining the integrity of the extracellular matrix and controlling the balance of articular cartilage homeostasis, thereby contributing to the deceleration of OA development (158, 159). In the pathological process of OA, cartilage degeneration is a key event. Research has shown that ferroptosis of chondrocytes exacerbates the progression of OA pathology. Both the induction of an inflammatory environment and a redox system imbalance promote ferroptosis in chondrocytes (160). Interleukin-1β (IL-1β), a factor that mimics an inflammatory condition, is capable of triggering alterations in the ROS concentrations within chondrocytes. It also enhances the accumulation of lipid peroxides and alters proteins associated with ferroptosis, such as GPX4, which ultimately result in the ferroptosis of chondrocytes (80, 160). Ferroptosis in chondrocytes increases the levels of MMP13 and IL-1β, simultaneously reducing the expression of type II collagen. This alteration in the balance of the extracellular matrix plays a role in the progression of OA (**Figure 4**).

Fer-1 activates the Nrf2 antioxidant system, leading to elevated GPX4 levels and increased type II collagen production in chondrocytes. Additionally, Fer-1 mitigates the alterations in ferroptosis-related protein expression induced by IL-1β (160). In an inflammatory setting induced by IL-1β, increased levels of hypoxia-inducible factor-2α (HIF-2α) coupled with diminished GPX4 expression in chondrocytes increase their susceptibility to ferroptosis (**Figure 4**) (80, 161). Antioxidant and anti-inflammatory substances, such as D-mannose, icaritin (162) and Fer-1, inhibit ferroptosis in chondrocytes by regulating the GPX4 pathway, thereby slowing cartilage degeneration and decelerating the pathological process of OA (80, 163).

Furthermore, subchondral bone is crucial for maintaining normal joint function. Ferroptosis may promote osteoclast bone resorption by inhibiting osteoblast function, thereby affecting the homeostasis and structural integrity of subchondral bone and leading to the occurrence of osteoarthritis (164, 165). Synovial inflammation promotes osteoarthritis. The synovium can secrete proinflammatory cytokines, leading to cartilage damage. Research has shown that excessive iron accumulation occurs in the synovium in haemophilic arthropathy, resulting in synovitis (150, 166). During the development of osteoarthritis, the synovium undergoes interstitial vascularization, fibrosis and hyperplasia (167), and macrophages and fibroblasts in the synovium can release proinflammatory cytokines, etc., which cause synovitis and cartilage degeneration.

RNA LINC00618 promotes apoptosis by increasing the levels of the pro-apoptotic protein, cleaved caspase-3, and also induces ferroptosis by inhibiting the cellular antioxidant system, suggesting a potential interaction between apoptosis and ferroptosis (168). Furthermore, cellular pyroptosis contributes to the progression of bone diseases. In the context of OA, the inflammatory response triggered by cellular pyroptosis exacerbates cartilage degeneration and synovial inflammation, thereby accelerating OA progression (169). These findings suggest that there may be mutual regulation between ferroptosis and other forms of cell death, warranting further investigation into the molecular interaction mechanisms to explore their potential clinical significance.

Epigenetic modifications play a crucial role in regulating ferroptosis in chondrocytes. The histone methyltransferase NSD1 has been identified as having potential therapeutic value by upregulating the H3K36me2 modification, thereby activating the expression of the transcription factor SOX9, which subsequently downregulates the key enzyme for ferroptosis, ACSL4, thus attenuating chondrocyte ferroptosis (170). Furthermore, the lactate metabolizing enzyme LDHB promotes H3K18 histone lactoylation, enhances ACSL4 expression, and induces chondrocyte ferroptosis, representing another epigenetic regulatory target for OA (171).

### 4.3 Ferroptosis in lumbar disc herniation

One of the most common reasons for chronic low back pain is lumbar disc herniation. After degenerative changes occur in the disc, the annulus fibrosus can break, leading to the protrusion or bulging of the nucleus pulposus, either independently or in conjunction with the annulus fibrosus and the cartilaginous plate. This condition can irritate or compress the spinal nerve roots, resulting in low back pain (172, 173).

Lumbar intervertebral disc protrusion has a high recurrence rate and is more prevalent in middle-aged and elderly individuals, with an increasing incidence among younger individuals. The intervertebral disc is a fibrocartilaginous structure situated between two adjacent lumbar vertebrae that serves to enhance spinal mobility, absorb shock, and safeguard the spinal cord. A tear in the annulus fibrosus, alongside the degeneration of the nucleus pulposus and the breakdown of the cartilage plate, play roles in the degeneration of the intervertebral disc, which can ultimately result in lumbar disc herniation.

**Figure 5:**
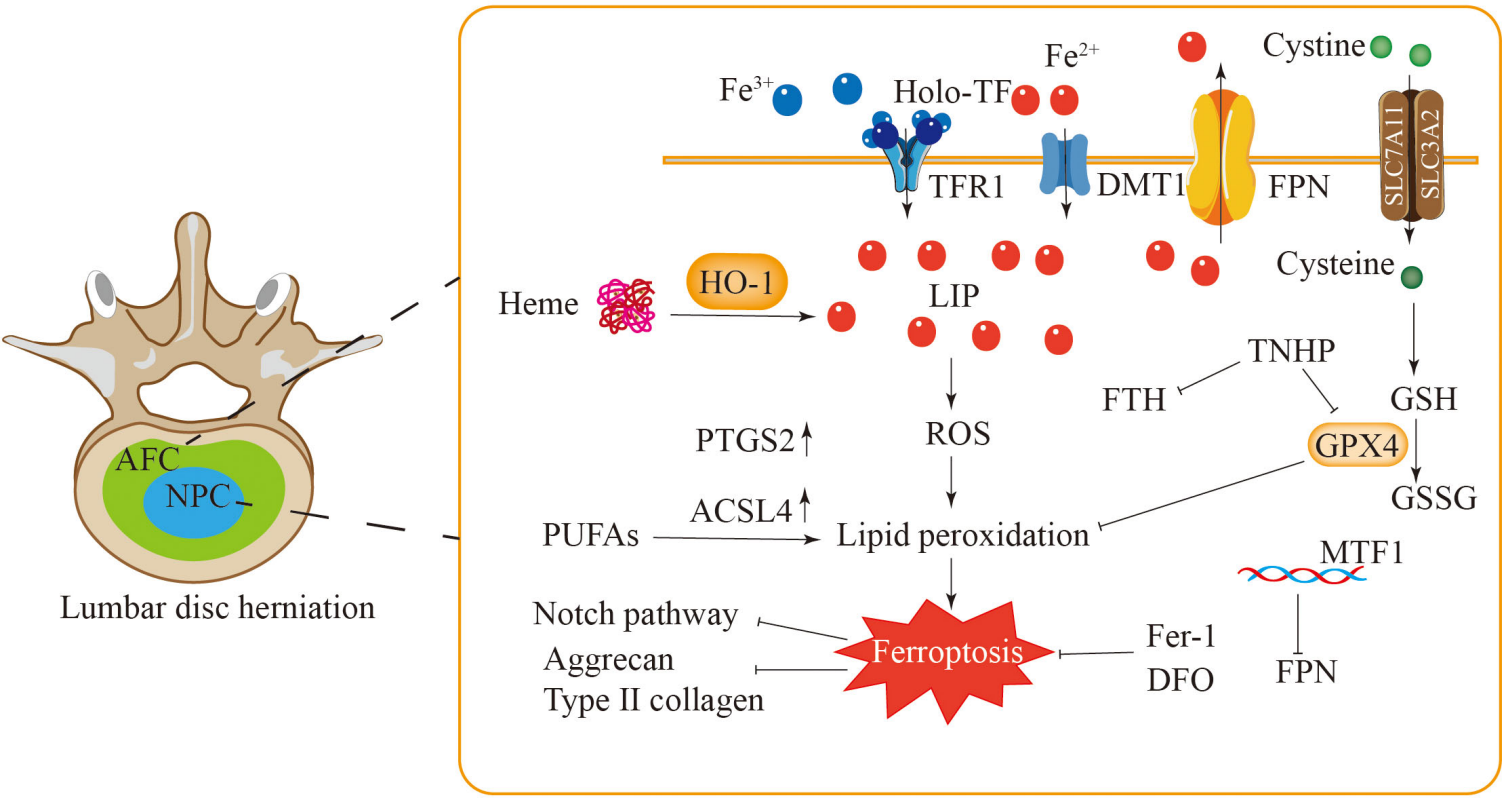
Ferroptosis is closely related to lumbar disc herniation. The levels of GPX4 and FTH in AFC and NPC of patients with lumbar disc herniation are decreased, while the expressions of PTGS2 and ACSL4 are increased, and these changes are inhibited by Fer-1 and DFO. In NPC, the elevated levels of HO-1 and iron intensify lipid peroxidation and induce ferroptosis in NPC. The ferroptosis of NPC may be related to the Notch signaling pathway. Additionally, MTF1 inhibits cell ferroptosis by regulating hepcidin. Ferroptosis in AFC and NPC leads to a decrease in type II collagen and aggrecan levels and extracellular matrix degradation.

Research has recognized ferroptosis as a possible pathogenic mechanism associated with lumbar disc herniation, although detailed mechanistic investigations remain scarce. Research indicates that rats suffering from lumbar degenerative disc disease exhibit reduced levels of the antioxidants GPX4 and FTH within the disc tissue. Conversely, the levels of cyclooxygenase (PTGS2) and ACSL4, both of which are integral to lipid metabolism, are elevated (174). These findings indicate the potential involvement of ferroptosis in the pathological mechanisms related to LDH. Furthermore, iron overload has been shown to exacerbate disc degeneration, ultimately contributing to herniation. In the course of the experiments, tert-butyl hydroperoxide (TBHP) was used to induce oxidative stress in annulus fibrosus cells (AFCs) and nucleus pulposus cells (NPCs). As the concentration of TBHP increased, the expression of FTH and GPX4 decreased, and the lipid ROS levels in the AFCs and NPCs increased. Characteristic features of ferroptosis, such as the crumpling of mitochondria and an increase in the mitochondrial membrane density, were observed at the mitochondrial level. Importantly, these alterations were attenuated by iron-induced death inhibitors (Fer-1 and DFO), further reinforcing the association between ferroptosis and the pathological mechanisms of LDH (**Figure 5**) (174, 175).

Neovascularization is another characteristic of intervertebral disc protrusion. An experiment demonstrated the presence of neoangiogenesis in the herniated nucleus pulposus, which may contribute to tissue damage (176). Compared with patients with normal nucleus pulposus, patients with lumbar disc herniation presented higher haemoglobin concentrations in the disc nucleus pulposus, a higher quantity of dark iron particles following staining, and elevated expression levels of HO-1 (177). Increased concentrations of haemoglobin and haem may trigger ferroptosis, potentially through a mechanism linked to the Notch signaling pathway (**Figure 5**) (177).

Atypical patterns of NPCs significantly contribute to the pathological progression of lumbar disc degeneration. Although these cells can produce components of the extracellular matrix, reductions in the levels of type II collagen and aggregated proteoglycans are often observed following disc injury (178). Furthermore, the iron overload and lipid peroxidation induced by TBHP in human NPCs can be significantly reversed by Fer-1 or the iron chelator DFO (176).

FPN is an important regulatory factor for cellular iron homeostasis. The overexpression of FPN can alleviate iron ion overload and ferroptosis in intervertebral disc cells. MTF1 eliminates intracellular iron overload by regulating FPN, thereby protecting intervertebral disc cells from ferroptosis (**Figure 5**) (179). Chondrocytes are essential for maintaining the proper function of intervertebral discs. Research has shown a notable decrease in the chondrocyte population in degenerated intervertebral discs (180), with iron overload being a key contributing factor. In the chondrocytes of the iron overload model, the levels of GPX4 and SLC7A11 within the antioxidant system were lower, whereas lipid peroxidation levels, indicated by 4-HNE, were higher, and their mitochondria exhibited characteristics similar to ferroptosis (175). These findings suggest that the reduction in the number of chondrocytes within degenerated intervertebral discs could be linked to ferroptosis.

Furthermore, a notable rise in the expression levels of genes associated with autophagy, as well as a higher quantity of autophagic vacuoles and lysosomes, has been detected in degenerated intervertebral discs and the annulus fibrosus (181, 182). This finding suggests a potential link between the pathological process of LDH and autophagy. Both autophagy and ferroptosis contribute to the pathological process of disc degeneration. Notably, ferritin autophagy enhances autophagy and ferroptosis in NPC and AFC, whereas Fer-1 inhibits this process (174). Therefore, further investigation into the interaction network between ferroptosis and autophagy in bone diseases may deepen our understanding of their pathogenesis and provide novel insights for the treatment of such conditions.

In LDH, the inhibition of the DNA methyltransferase DNMT3B effectively prevents the onset of ferroptosis in NPCs and enhances cellular activity and mitochondrial function, suggesting a critical role in the regulation of iron homeostasis (183). Furthermore, TBHP can promote the accumulation of iron ions and induce ferroptosis by inhibiting the expression and nuclear export of the FPN. This process is regulated by the JNK/MTF1/FPN pathway, which constitutes a potential therapeutic target (179).

### 4.4 Ferroptosis in osteosarcoma

Osteosarcoma is a cancerous tumor of the bone characterized by high mortality rates, significant disability, and frequent metastasis and recurrence, primarily affecting the bones and lungs (184). The incidence rate is the highest among children and adolescents, with a higher rate in males than in females. OS often occurs near the growth plates of long bones (185). Clinically, the main treatment approach combines surgery with radiotherapy and chemotherapy. However, the effectiveness of traditional treatment methods for osteosarcoma is still limited, as the 5-year survival rate falls within the range of 60% to 70%. Moreover, significant progress in conventional therapies has been scarce over the past few years.

**Figure 6:**
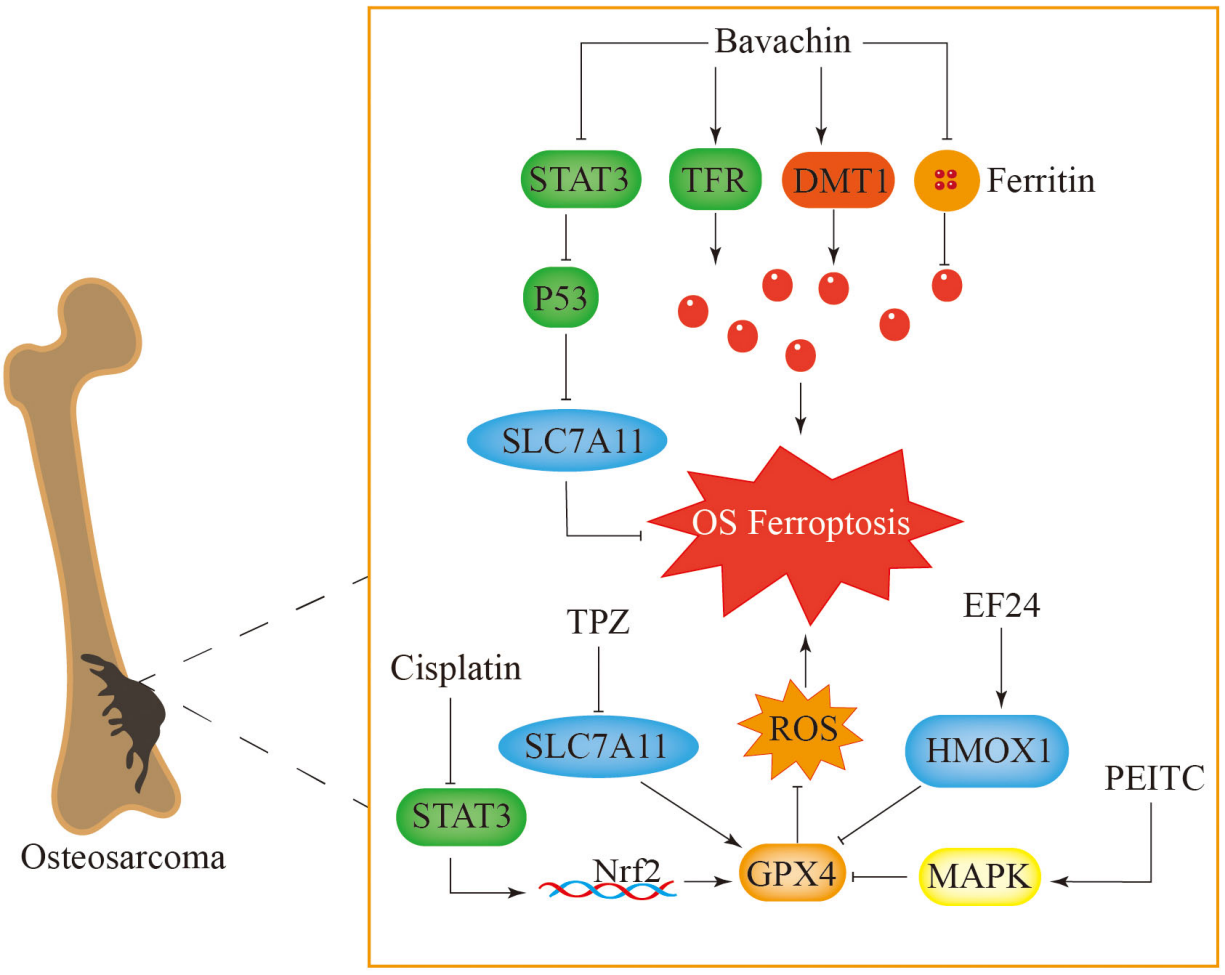
Ferroptosis plays a key role in the treatment of osteosarcoma. Bavachin induces ferroptosis in osteosarcoma cells by increasing intracellular iron levels and inhibiting the STAT3-P53-SLC7A11 pathway. TPZ induces ferroptosis in osteosarcoma cells by inhibiting the expression of SLC7A11 in the classical ferroptosis signaling pathway. Cisplatin causes cell ferroptosis through the STAT3-Nrf2-GPX4 signaling pathway. PEITC and EF24 respectively activate the MAPK and HMOX1 signaling pathways, inhibit GPX4, cause accumulation of reactive oxygen species in cells, and induce ferroptosis.

Ferroptosis has been recognized as a crucial element in the progression of tumors and the immune response, among other biological processes (186). Bavachin exerts its anticancer effects by triggering ferroptosis in osteosarcoma cells through multiple mechanisms. Among them, a reduction in matrix metalloproteinases (MMPs) leads to ferroptosis-like features in mitochondria (187). Furthermore, bavachin upregulated the expression of the transferrin receptor and DMT1 while downregulating the expression of FTL and FTH. These changes resulted in elevated intracellular Fe^2+^ levels and the downregulation of p-STAT3, SLC7A11, and GPX4 levels (57). Thus, bavachin clearly induces ferroptosis in osteosarcoma cells by modulating genes and proteins associated with cell death. Notably, the upregulation of P53 expression leads to lower expression levels of SLC7A11 and GPX4, promoting the accumulation of ROS and the lipid peroxidation product malondialdehyde (MDA), which in turn induces ferroptosis in osteosarcoma. This process is inhibited by ferroptosis inhibitors (**Figure 6**) (187).

Tirapazamine (TPZ) is a novel anticancer drug that exerts its antitumour effect under hypoxic conditions (188). Under hypoxic conditions, SLC7A11 expression is reduced, whereas increases in MDA and Fe^2+^ levels increase ROS accumulation in osteosarcoma cells. This process triggers ferroptosis and suppresses the proliferation and metastasis of osteosarcoma (189). Cisplatin is a commonly used drug for treating osteosarcoma, but osteosarcoma cells can develop resistance to it, which reduces its therapeutic effect (190). Measurements of ferroptosis-associated proteins in osteosarcoma cells indicate that compared with normal cells, drug-resistant cells exhibit elevated GPX4 levels. Moreover, the use of ferroptosis inhibitors increases the sensitivity of these osteosarcoma cells to cisplatin, while diminishing the levels of ferroptosis-related proteins further enhances this sensitivity (191). The signaling pathway involving STAT3/Nrf2/GPX4 is integral to the resistance observed in osteosarcoma cells and may serve as a promising therapeutic target to improve the effectiveness of cisplatin against bone tumors (**Figure 6**) (191).

Phenethyl isothiocyanate (PEITC) can cause cell cycle arrest and trigger apoptosis in tumor cells. It promotes ferroptosis in bone tumor cells by inducing oxidative stress, depleting GPX4, increasing ROS levels, and activating the MAPK signaling pathway (192, 193). The synthetic derivative of curcumin biphenyl-difluoroketone (EF24) has been reported to trigger cell apoptosis, restrain cell growth, and diminish metastatic capabilities (194, 195). Furthermore, EF24 induces ferroptosis in osteosarcoma cells by increasing the levels of lipid peroxides and intracellular iron ions. It also achieves this effect by increasing the expression of haem oxygenase 1 (HMOX1) and reducing the expression of GPX4 (196). This process can be counteracted with the application of ferroptosis inhibitors.

In recent advances in cancer therapy, it has been found that there may be an interaction between the molecular mechanisms involved in ferroptosis and cellular pyroptosis (197). Anti-tumor immune cells, such as T cells, can induce tumor cells to undergo pyroptosis while simultaneously increasing ROS to trigger ferroptosis, thereby exerting anti-tumor effects (198, 199). However, the molecular interactions between ferroptosis and pyroptosis remain unclear and warrant further investigation.

Induction of cellular ferroptosis significantly enhances the sensitivity of OS cells to both radiotherapy and chemotherapy. Although the specific epigenetic regulatory mechanisms remain incompletely understood, evidence suggests that epigenetic factors, including miRNAs and RNA methylation, may play a role in the therapeutic process by regulating key genes associated with ferroptosis, such as GPX4 and SLC7A11 (200).

## 5 Therapeutic perspectives and challenges

Ferroptosis is closely related to orthopaedic diseases, and inhibiting ferroptosis in patients with related orthopaedic diseases may constitute a new treatment method. Ferroptosis inducers can induce ferroptosis through different targets. Among these compounds, erastin, sulfasalazine (SAS), RSL3, sorafenib, and FIN56 induce ferroptosis through the inhibition of the GSH/GPX4 pathway (201, 202). t-BuOOH triggers ferroptosis by modulating cellular lipid metabolism (203), whereas erastin and RSL5 induce ferroptosis by altering cellular iron metabolism (204). Temozolomide (TMZ) promotes ferroptosis by upregulating DMT1 (205). FIN56 can also induce ferroptosis in cells by depleting CoQ (52).

In addition, many ferroptosis inhibitors suppress ferroptosis in cells through a series of pathways. The consumption of excessive iron ions in cells is a direct method to inhibit ferroptosis. Compounds that bind iron, such as ciclopirox olamine (CPX), DFO, and deferiprone (DFP), inhibit ferroptosis by chelating iron ions and subsequently reducing ROS production (3, 206, 207). The accumulation of excess lipid ROS in cells is a central feature of ferroptosis. Fer-1, liproxstatin-1, α-tocopherol, VKH2 and other substances inhibit ferroptosis by suppressing lipid peroxidation (208, 209). The GSH/GPX4 pathway serves as a crucial antioxidant mechanism within cells. β-Mercaptoethanol (β-ME) and selenium (Se) are capable of increasing the activity of the GSH/GPX4 pathway via distinct mechanisms, which in turn suppress ferroptosis (50, 210). In addition, prominin 2, miR-522, iNOS, and other molecules inhibit ferroptosis through different pathways (**Table 1**) (27, 263).

**Table 1:** The mechanism of ferroptosis-related targets in bone and joint diseases.

Bone and joint diseases	Drug	Ferroptosis-related targets	Study subject	Mechanism	Reference
Osteoporosis	Fer-1, ZnPP	HO-1, GPX4	Male C57BL/6J mice	Reduce the expression of HO-1 and increase the expression of GPX4 to restore the redox balance within the cells	(124)
	CSE	Lipid ROS	Male SD rat	The AMPK signaling pathway in the cells was activated, which further promoted ferroptosis mediated by NCOA4, resulting in the deposition of lipid peroxidation	(211)
	Mangiferin	Nrf2, SLC7A11, GPX4	C57BL/6 mice	Mangiferin inhibits ferroptosis by activating Nrf2, increasing the expression of SLC7A11 and GPX4, and reducing the level of 4-HNE	(212)
	SGI-1027	GPX4	C57BL/6 J mice	SGI-1027 enhances GPX4 expression and reduces lipid peroxidation marker MDA in cells, thereby alleviating ferroptosis	(213)
	Maresin1	Nrf2, GPX4, SLC7A11	MC3T3-E1 cells, male SD rats	MaR1 alleviates ferroptosis by activating the Nrf2 pathway	(214)
	Aconine	GPX4, ACSL4	Female C57/BL6 mice	Aconine can inhibit the NF-κB signaling pathway, thereby suppressing GPX4 and upregulating ACSL4	(215)
	Deferoxamine, Fer-1	GPX4, SLC7A11	MC3T3-E1 cells, female C57/BL6 mice	DFO and Fer-1 increase the expression of GPX4 and SLC7A11 to inhibit ferroptosis	(216)
	Poliumoside	Lipid ROS	Male C57BL/6 mice	Poliumoside inhibits ferroptosis by activating the Nrf2/GPX4 pathway	(217)
	Melatonin	Nrf2/HO-1 pathway	MC3T3-E1 cells, SD rats	Melatonin Protected MC3T3 Cells against Ferroptosis via the Nrf2/HO-1 Signaling Pathway	(134)
	AVI	GPX4	Male C57BL/6J mice	AVI can enhance the expression of GPX4 to reduce ferroptosis	(218)
	VK2	AMPK/SIRT1 pathway	Male C57BL/6J mice	VK2 inhibits ferroptosis by activating the AMPK/SIRT1 signaling pathway	(219)
	Pantethine	PSMD14	C57BL/6 mice	Pantethine can restore the expression of SLC7A11 in cells	(220)
	Fraxin	Nrf2/GPX4 pathway	Male C57BL/6J mice	Fraxin inhibits ferroptosis by activating the Nrf2/GPX4 signaling pathway	(221)
	Metformin	GPX4, FTH1, SLAC7A11	SD rats, MC3T3-E1 cells	Metformin activates the AMPK/Nrf2 pathway to inhibit ferroptosis	(222)
	QEP	ATM, AKT/PI3K pathway	hFOB 1.19 cells	QEP inhibits ferroptosis by down-regulating ATM and activating the AKT/PI3K signaling pathway	(223)
	FLL	GPX4, SLAC7A11	SD rats	FLL upregulates the expression of GPX4 and SLAC7A11 to inhibit ferroptosis	(224)
	Aucubin	Lipid ROS	HBMSCs, female SD rats	Aucubin reduces the level of ROS and protects cells from ferroptosis	(225)
	Berberine	4-HNE, PTGS2, FTH, TF	Male C57BL/6 mice	Berberine inhibits the levels of PTGS2, TF and 4-HNE, and increases the level of FTH to inhibit ferroptosis	(226)
Osteoarthritis	VK2	GPX4	Male SD rats	VK2 can increase the level of GSH and reduce the content of MDA, thereby inhibiting ferroptosis	(227)
	Biochanin A	Ferrous ion	Male C57BL/6 mice	Biochanin A reduces cellular iron levels by inhibiting TFR1 and promoting ferroprotein to suppress ferroptosis	(228)
	Baicalein	AMPK, Nrf2	C57BL/6J (WT) mice, AMPKα-KO mice	Baicalein inhibits ferroptosis of chondrocytes by enhancing the activity of the AMPK/Nrf2/HO-1 signaling pathway	(229)
	Melatonin	NOX4	Female C57BL/6 mice	Melatonin inhibits the expression of NOX4 on mitochondria, alleviates mitochondrial dysfunction, and thereby suppresses ferroptosis	(230)
	D-mannose	HIF-2α	C57BL/6 J mice	D-mannose decreases chondrocyte ferroptosis sensitivity via inhibiting HIF-2α expression	(80)
	Deferoxamine	Lipid ROS, Nrf2	Male C57BL/6 mice	DFO reduced the accumulation of lipid ROS and MDA, and activated the Nrf2 antioxidant system, thereby alleviating ferroptosis	(231)
	ICA	SLC7A11, GPX4	SW1353 cells, male SD rats	ICA alleviated ferroptosis of chondrocytes by enhancing SLC7A11/GPX4 signaling	(232)
	PCA NPs	Lipid ROS, GPX4, SLC7A11	Female SD rats	PCA NPs increase the expression of GPX4 and SLC7A11 and reduce the level of lipid ROS in cells, thereby inhibiting ferroptosis	(233)
	Metformin	AMPK/ACC pathway	Male C57BL/6J (WT) mice	Metformin reshapes lipid availability through the AMPK/ACC pathway and reduces the ferroptosis sensitivity of cells	(234)
	Sar	YAP1	Male SD rats	Sar increases the expression of YAP1, thereby reducing the sensitivity of cells to ferroptosis	(235)
	ATX	Lipid ROS, mitochondrial iron	Male SD rats	ATX can inhibit lipid ROS levels and regulate mitochondrial function, thereby suppressing ferroptosis	(236)
	Quercetin	SIRT1/Nrf-2/HO-1 pathway	SD rats	Quercetin alleviates cellular oxidative damage and inhibits ferroptosis through the SIRT1/Nrf-2/HO-1 pathway	(237)
	Vinpocetine	Nrf2, GPX4	C57BL/6 mice	Vinpocetine activates the Nrf2/GPX4 pathway and inhibits ferroptosis	(238)
	Curcumin	Ferrous ion, lipid ROS	BALB/C mice	Curcumin upregulated the expression level of Nrf2, reduced cellular lipid ROS and cellular iron levels, thereby inhibiting ferroptosis of cells	(239)
	Plumbagin	MAPK	Male SD rats	Plumbagin inhibits ferroptosis of cells by regulating the MAPK signaling pathway	(240)
	OI	ROS, GPX4	Male C57BL/6J mice	OI inhibits ferroptosis by reducing the level of ROS in cells and maintaining the expression of GPX4	(241)
	Rosiglitazone	ACSL4	Male C57BL/6 mice, CP-M087 cells	Rosiglitazone inhibits cellular ferroptosis by targeting ACSL4	(242)
	Sappanone A	Nrf2, SIRT1, GPX4	Male SD rats	Sappanone A activates the Nrf2 signaling pathway by targeting SIRT1, and increases the expression of GPX4, thereby inhibiting ferroptosis of cells	(243)
	Paeonol	ACSL4	Male C57BL/6 mice	Paeonol protects chondrocytes from ferroptosis by inhibiting ACSL4	(244)
LDH	PDA NPs	ROS, Ferrous ion, FHC	293T cells, SD rats	PDA NPs protect cells from ferroptosis by reducing cellular iron overload, regulating the expression of FHC, and eliminating phospholipid hydroperoxides	(245)
	Cynarin	Ferrous ion, Nrf2, GPX4	Male SD rats	Cynarin inhibits ferroptosis by increasing the expression of GPX4 and Nrf2, thereby suppressing the increase of cellular iron and lipid ROS	(246)
	NDGA	Nrf2	SD rats	NDGA may promote the expression of Nrf2, increase the level of GPX4 in cells, thereby inhibiting ferroptosis	(247)
	Fisetin	Nrf2/HO-1 pathway	SD rats	Fisetin may inhibit ferroptosis by suppressing cellular oxidative stress through the Nrf2/HO-1 pathway	(248)
	Melatonin	Ferrous ion	C57BL/6 mice	Melatonin inhibits cellular iron overload, thereby protecting cells from ferroptosis	(249)
	Tinoridine	Nrf2	Male SD rats	Tinoridine exerts its inhibitory effect on ferroptosis through Nrf2	(250)
	FZD	NF-κB	Male SD rats	FZD inhibits ferroptosis by suppressing the NF-κB signaling pathway	(251)
	GA	Nrf2, lipid ROS	Male SD rats	GA alleviates ferroptosis by reducing lipid ROS in cells	(252)
	Aspirin	GPX4	Male SD rats	Aspirin stabilizes the targeting effect of GPX4, thereby inhibiting ferroptosis	(253)
	Hesperidin	Nrf2, NF-κB	Male C57BL/6 mice	Hesperidin may inhibit ferroptosis by enhancing the expression of Nrf2 and suppressing the NF-κB pathway	(254)
	Tomatidine	Nrf2/HO-1/GPX4 pathway	Male C57BL/6 mice	Tomatidine inhibits ferroptosis in cells by activating the Nrf2/HO-1/GPX4 pathway	(255)
Osteosarcoma	Baicalin	Nrf2, GPX4	MG63 and 143B cells, BALB/c-nude mice	Baicalein physically interacts with Nrf2 and inhibits the expression of GPX4, thereby promoting ferroptosis	(256)
	Butyrate	SLC7A11	BALB/c nude mice	Butyrate downregulates the transcription of SLC7A11, thereby promoting cellular ferroptosis	(257)
	Sulforaphane	SLC7A11	143B and SJSA-1 cells, BALB/c nude mice	Sulforaphane induces ferroptosis by influencing the level of SLC7A11 in cells	(258)
	EF24	GPX4, HMOX1	U2os and Saos-2 cells	EF24 upregulated HMOX1 to suppress GPX4 expression to induce ferroptosis by increasing MDA level, ROS level and intracellular ferric ion level	(196)
	Casticin	HMOX1, Ferrous ion	143B cells, MG63 cells, BALB/c nude mice	Casticin induces ferroptosis in cells through HMOX1-mediated iron overload	(259)
	ZOL	POR, ROS	MG63 cells, 143B cells, female BALB/c Nude mice	ZOL increases the expression of POR, thereby raising the levels of cellular ROS and lipid peroxidation, and thereby inducing ferroptosis in cells	(260)
	Penfluridol	ROS, Ferrous ion, GSH	MG63 cells, 143B cells	Penfluridol induces ferroptosis by increasing intracellular iron levels and reducing GSH levels	(261)
	Artesunate	TFR, DMT1, NCOA4	MG63 cells, 143B cells	Artesunate upregulates the expression of TFR and DMT1 and triggers ferritin autophagy by upregulating the expression of NCOA4, thereby increasing cellular iron levels and inducing ferroptosis	(262)
	Tirapazamine	SLC7A11	MNNG/Hos cells, 143B cells, U2OS cells	Tirapazamine induces ferroptosis in cells by inhibiting SLC7A11	(189)
	Bavachin	STAT3/P53 /SLC7A11 pathway	MG63 cells, HOS cells	Bavachin down-regulates the expression of SLC7A11 and GPX4, and induces ferroptosis in cells through the STAT3/P53/SLC7A11 pathway	(187)

Fer-1, ferrostatin-1; ZnPP, zinc protoporphyrin IX; HO-1, heme oxygenase-1; ACSL4, Acyl-CoA synthetase long-chain family member 4; CSE, cigarette smoke extract; Nrf2, nuclear factor-erythroid 2-related factor 2; AVI, asperosaponin VI; VK2, vitamin K2; FTH1, ferritin heavy chain 1; SLC7A11, solute carrier family 7, member 11; TFR1, transferrin receptor 1; QEP, Qing’e Pill; FLL, Fructus Ligustri Lucidi; 4-HNE, 4-hydroxynonenal; PTGS2, prostaglandin-endoperoxide synthase 2; TF, transferrin; FTH, ferritin heavy chain; MDA, malondialdehyde; DFO, deferoxamine; ICA, Icariin; LDH, lumbar disc herniation; PCA NPs, poly(p-coumaric) nanoparticles; Sar, Sarsasapogenin; YAP1, Yes-associated protein 1; ATX, astaxanthin; OI, 4-octyl Itaconate; ROS, reactive oxygen species; FHC, ferritin heavy chain; NDGA, nordihydroguaiaretic acid; FZD, Fuzi decoction; GA, gallic acid; SD, Sprague-Dawley; ZOL, zoledronic acid; POR, P450 oxidoreductase; GSH, Glutathione; DMT1,divalent metal transporter 1; TFR, transferrin receptor protein; NCOA4, nuclear receptor coactivator 4.

Many studies efforts have documented a strong link between cellular ferroptosis and bone health, indicating that manipulating cellular ferroptosis may provide new opportunities for addressing related bone and joint conditions. In osteoporosis, ferroptosis contributes to the pathological process of the disease by altering the functions of osteoblasts, osteoclasts, and mesenchymal stem cells. Notably, HO-1 and mitochondrial ferritin play critical roles in disease progression, and inhibiting their overexpression may represent a novel management strategy for osteoporosis. Furthermore, ferroptosis exacerbates the progression of OA. Inhibitors of ferroptosis, such as Fer-1, D-mannose, and icariin, may mitigate the pathological processes of OA by suppressing ferroptosis in chondrocytes. Ferroptosis may be one of the pathogenic mechanisms of lumbar intervertebral disc protrusion. The ferroptosis inhibitor Fer-1 and the iron chelators DFO and MTF1 can protect intervertebral disc cells from ferroptosis. Ferroptosis plays a crucial role in the pathological progression of osteosarcoma, and agents such as bavachin, TPZ, cisplatin, PEITC, and EF24 induce ferroptosis in osteosarcoma cells, thereby exerting therapeutic effects (**Table 1**).

**Figure 7:**
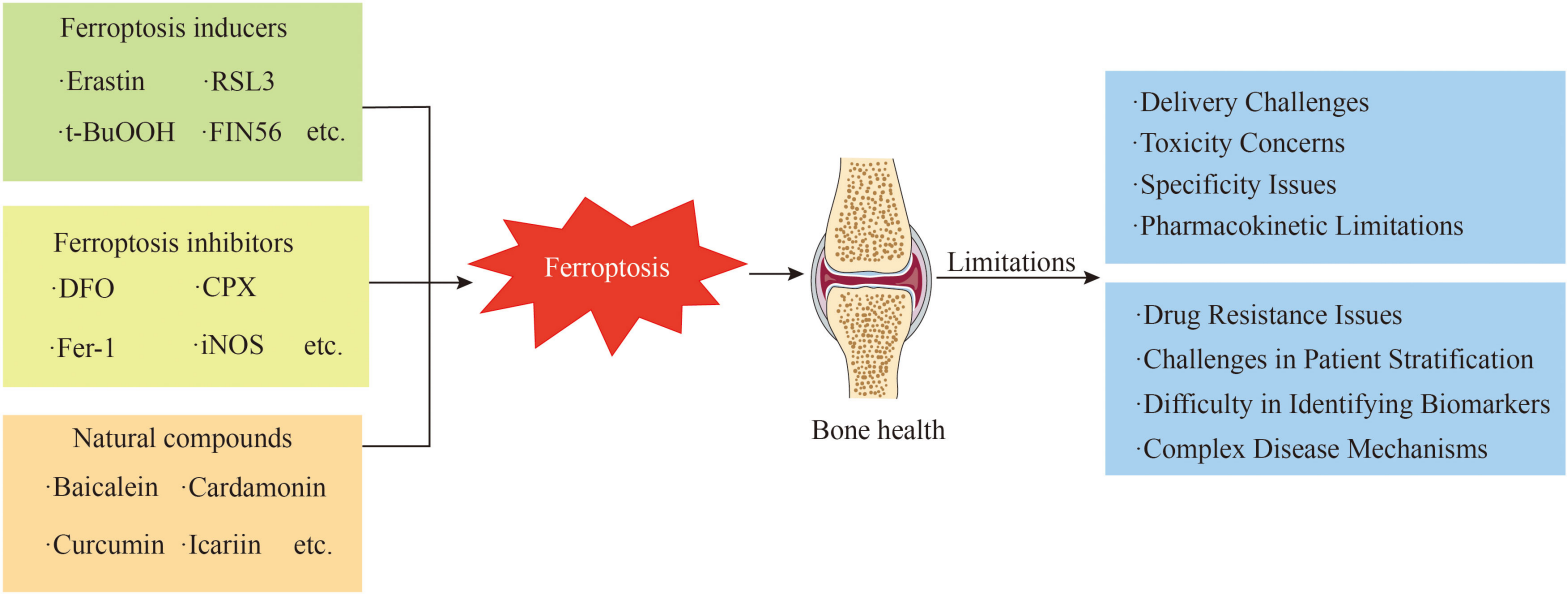
This figure presents the multiple challenges faced in the clinical transformation of iron-addicted drugs.

Ferroptosis inducers and inhibitors exhibit limitations concerning their specificity and delivery mechanisms. Some inducers promote ferroptosis by inhibiting the GSH/GPX4 pathway (3); however, this pathway is implicated in various physiological and pathological processes, suggesting that the specific induction of ferroptosis may inadvertently disrupt normal cellular functions (264). Regarding delivery, most ferroptosis inducers are small-molecule compounds with diverse chemical properties and stability profiles, complicating their efficient delivery to target cells or tissues. These compounds are prone to rapid metabolism or degradation, leading to inadequate induction in specific tissues or potential toxicity in non-target sites. Similarly, many large-molecule ferroptosis inhibitors struggle to penetrate cell membranes effectively, hindering their ability to exert intracellular effects. Prolonged use of ferroptosis inhibitors may result in toxic accumulation. For instance, extended use of high-dose iron chelators can induce iron deficiency in the body, adversely impacting hematopoietic function, while overdosing on certain antioxidant-based inhibitors may impair coagulation function (58). Some ferroptosis-targeting agents have poor pharmacokinetic properties, such as low solubility, high metabolic clearance, low cellular permeability, and short half-lives (**Figure 7**).

The pathogenesis of OP, OA, and OSinvolves multiple factors and signaling pathways. While ferroptosis plays a role in these diseases, it is not the sole mechanism. For example, OP is influenced by factors such as bone metabolism, hormonal regulation, and genetic predisposition. OA involves joint cartilage degeneration, inflammation, and immune responses. OS is characterized by complex genetic mutations and tumor microenvironments. The interplay between ferroptosis and other mechanisms in these diseases requires further research to determine the optimal strategies for targeting ferroptosis in therapy (265).

Identifying reliable biomarkers to monitor ferroptotic activity *in vivo* and predict treatment efficacy is challenging. Without specific biomarkers, it is difficult to evaluate the therapeutic effects of ferroptosis-targeting agents in clinical trials and adjust treatment plans accordingly. This complicates the precise application of these agents in treating OP, OA, and OS (266). There is significant heterogeneity among patients with OP, OA, and OS. Identifying patient subgroups that are sensitive to ferroptosis-targeting agents and determining the optimal treatment populations remain unresolved issues. This limits the widespread application of ferroptosis-targeting therapies in clinical practice. Similar to other anticancer therapies, ferroptosis-targeting agents may face drug resistance challenges in OS treatment. Tumor cells can develop adaptive mechanisms to resist ferroptosis induction, such as upregulating antioxidant systems or altering iron metabolism pathways. This reduces the efficacy of ferroptosis-targeting agents over time and limits their long-term therapeutic potential (267).

Translating these findings into effective therapies for OP, OA, and OS presents numerous challenges. In OP treatment, there is a notable absence of a single drug that possesses high specificity and efficacy alongside a favorable safety profile. Therefore, exploring combinations of different drugs to achieve synergistic effects while minimizing side effects is essential (268). For OA, the targeted delivery of drugs to affected tissues, such as articular cartilage and synovium, is crucial; however, the current delivery systems struggle to achieve precise targeting, negatively influencing therapeutic outcomes and increasing systemic adverse effects. In OS treatment, the heterogeneity of OS tumors results in variable responses to ferroptosis inducers, with some cells demonstrating resistance. This necessitates extensive research to overcome drug resistance and identify novel strategies.

## 6 Conclusions

A new form of cell death, termed ferroptosis, was initially characterized in 2012 as distinct from apoptosis and autophagy. Since then, it has become a key area of interest in life science research. Ferroptosis is crucial for regulating bone equilibrium and regeneration. The overaccumulation of iron ions and intracellular oxidative stress are strongly linked to cartilage degeneration and impaired bone metabolism.

The connections between ferroptosis and bone and joint diseases involve mainly iron metabolism, ROS, GPX4, and lipid peroxidation processes. Inhibiting ferroptosis can safeguard osteoblasts from this particular form of cell death, consequently minimizing bone loss and mitigating osteoporosis. Moreover, it can also restrain ferroptosis in chondrocytes, thus delaying the progression of osteoarthritis. An increase in ferroptosis can lead to the death of osteosarcoma cells, suggesting a potential therapeutic approach for osteosarcoma. Various modulators of ferroptosis, including inhibitors and inducers, provide novel perspectives for addressing bone and joint disorders. In addition, our understanding of the iron cycling imbalance and ferroptosis in the pathological context of bone and joint diseases is lacking. The fundamental mechanisms and associated signaling pathways warrant further investigation. Currently, the exploration of ferroptosis in bone and joint diseases has focused mainly on cell and animal models, and future research should pay more attention to clinical studies.
